# Performance Evaluation of a Modular Detector Unit for X-Ray Computed Tomography

**DOI:** 10.3390/s130405167

**Published:** 2013-04-18

**Authors:** Zhe Guo, Zhiwei Tang, Xinzeng Wang, Mingliang Deng, Guangshu Hu, Hui Zhang

**Affiliations:** Department of Biomedical Engineering, Tsinghua University, Beijing 100084, China; E-Mails: guozhe06@gmail.com (Z.G.); tangzhiwei@wandong.com.cn (Z.T.); wxzfly@126.com (X.W.); deng8803@126.com (M.D.); hgs-dea@tsinghua.edu.cn (G.H.)

**Keywords:** computed tomography, X-ray detector, performance evaluation, uniformity correction

## Abstract

A research prototype CT scanner is currently under development in our lab. One of the key components in this project is the CT detector. This paper describes the design and performance evaluation of the modular CT detector unit for our proposed scanner. It consists of a Photodiode Array Assembly which captures irradiating X-ray photons and converts the energy into electrical current, and a mini Data Acquisition System which performs current integration and converts the analog signal into digital samples. The detector unit can be easily tiled together to form a CT detector. Experiments were conducted to characterize the detector performance both at the single unit level and system level. The noise level, linearity and uniformity of the proposed detector unit were reported and initial imaging studies were also presented which demonstrated the potential application of the proposed detector unit in actual CT scanners.

## 1. Introduction

X-ray computed tomography (CT) is a medical imaging technology that utilizes computer-processed X-ray projections to produce tomographic images or “slices” of specific areas of the body. These images are used for diagnostic and therapeutic purposes in various medical applications. CT was invented in 1972 by Godfrey Hounsfield and the first clinical CT images were produced at the Atkinson Morley Hospital in London in 1972 [1]. The original systems were dedicated to head imaging [1], and “whole body” systems with larger patient openings became available in 1976 [1]. CT imaging is widely used in the diagnosis of head, lung, cardiac, abdominal and pelvic diseases. An estimated of 72 million scans were performed in the United States in 2007 [2].

Because of the high system complexity and material cost, CT scanners are only available from a few manufacturers including Siemens, GE, Philips and Toshiba. Despite the development of CT technology, there are very limited choices in terms of CT components and instruments for the research community. In our lab, we proposed to build a modular CT scanner for the research purposes. One of the key components in this project is the CT detector. This paper describes the design and development of the modular CT detector unit for our proposed scanner. Evaluations were conducted to characterize the detector performance and results obtained in these experiments are reported. Initial imaging studies were also presented to demonstrate the functionality of the modular CT detector unit.

## 2. Materials and Methods

### 2.1. Modular Detector Unit

**Figure 1.**
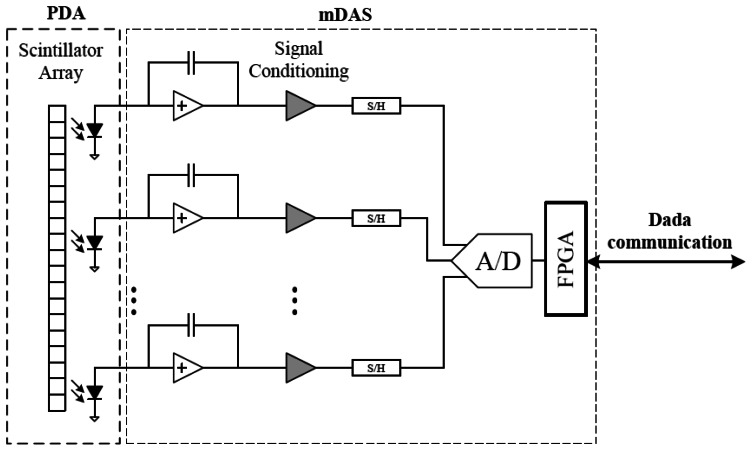
Illustration diagram of the detector unit.

**Figure 2.**
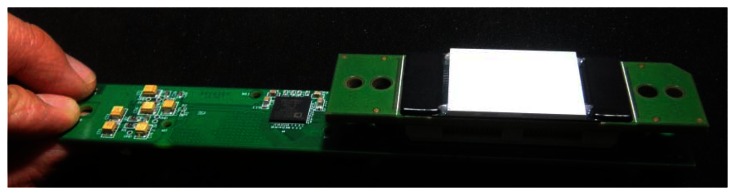
Photo of the modular detector unit.

Figure 1 shows the functional diagram of the proposed modular CT detector unit. It consists of a Photodiode Array Assembly (PDA) and a mini Data Acquisition System (mDAS). The PDA captures irradiating X-ray photons and converts the energy into electrical current. The mDAS mainly performs current integration and converts the analog signal into digital samples and output to follow-on circuits for further processing and data transfer. In our design, the ADAS1128 from Analog Device (ADI) is used to facilitate the development of mDAS, which is a highly integrated 128-channel, simultaneous sampling and current-to-digital converter ASIC [3]. The mDAS is directly attached to the back of the PDA through a high density connector. Figure 2 shows a photo of the modular detector unit.

Table 1 lists the main characteristics of the PDA used in our design. The PDA consists of a stacked scintillator array and photodiode array. The scintillator material is gadolinium oxysulfide (Gd_2_O_2_S) with a peak wavelength of 512 nm. The scintillator emits light photons when irradiated by X-ray photons. These light photons are then converted to electrical charges in the readout photodiode array. The photodiode array size is organized as 24 × 24 (channels × segments) with 16 small pixels in the middle and four large pixels on each side along the segment direction. The pitch size is 0.95 mm and 1.90 mm for the small pixels and large pixels, respectively.

**Figure 3.**
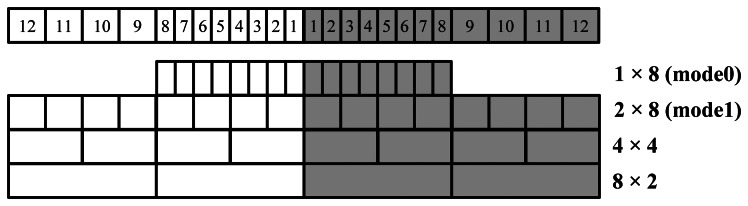
Detector unit readout modes.

The proposed detector unit supports variable pixel size with several readout modes and Figure 3 shows four of such modes. As shown in the figure, the detector outputs the 16 small pixel segments in the middle in mode 0, while in other mode, specific photodiode pixels are combined together as one readout segment. For example, in mode 1, every two small pixels in the middle are combined as one large pixel, together with the eight large pixels on the two sides, the detector outputs 16 large pixel segments. The detector also supports a float mode which disconnects the PDA from the mDAS, which is intended for test purposes. It is worthwhile to note that although the PDA is organized as a 24 × 24 array, a maximum of 16 segment outputs along the channels are supported so that the detector unit has a maximum of 384 channel outputs, such as in the mode 0 and mode 1.

### 2.2. Experiment Setup

**Figure 4.**
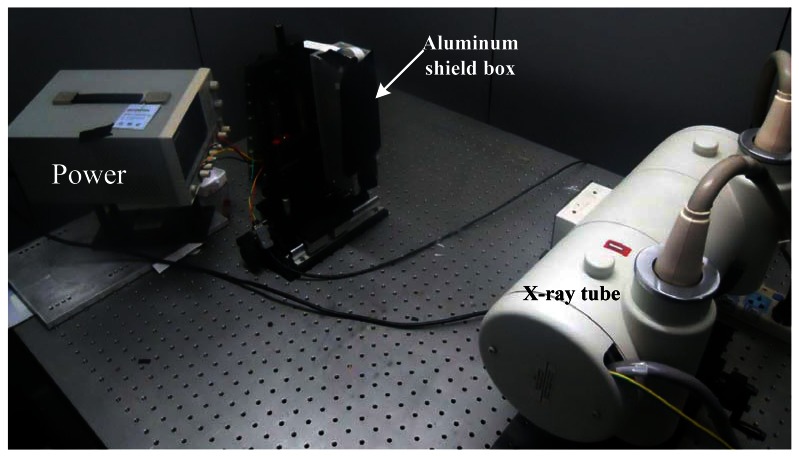
Photo of the modular detector unit.

The performance of the proposed detector unit was characterized at both the unit level and the system level. All the experiments were conducted in a lead-shielded and light tight room. Figure 4 shows the test setup using a single detector unit. The detector unit was placed in an aluminum box which acts as the shield for the detector unit. The lid of the aluminum box was removed for minimum attenuation of the irradiating X-ray photons. In order to make a dark environment for the detector unit, the aluminum box opening was sealed with black light-tight tapes. The aluminum box was mounted on a moving stage for vertical alignment of the detector unit to the focal spot of the X-ray tube (model G297 from Varian). An Indico 100 X-ray generator from CPI was used to control the generation of X-ray photons during the tests. In order to read out data from the detector unit, a custom designed circuit board was made which converts the data output from the detector unit into RS232 protocol and transfers the data to a host PC, and a MATLAB GUI tool was developed on the host PC to receive data from the detector unit and store as files for further analysis.

**Figure 5.**
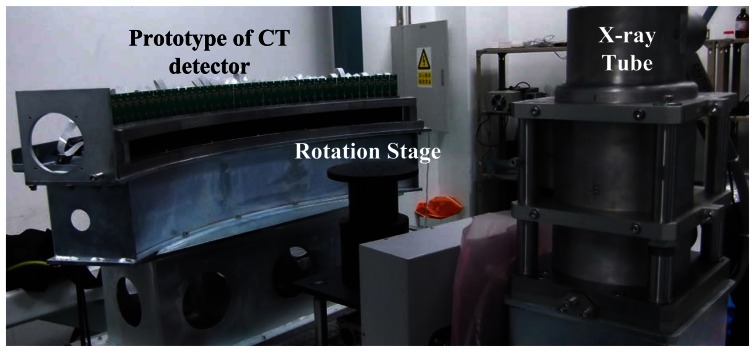
Setup of the CT detector tests.

Figure 5 shows the test setup of a prototype CT detector system. The detector system consists of 40 detector units mounted side by side along an arc rail. The detector system was installed on an optical table with the arc center points to the focal spot of an X-ray tube. A high speed rotation stage was placed between X-ray tube and detector, which was used to mount imaging objects. The setup was used to simulate the gantry rotation in an actual CT scanner. A custom designed data acquisition control and management system was developed to read out the data from the CT detector and transfer the data to a host PC through a 1.25 Gbps optical fiber. The host PC installs a custom made data communication board to receive data from the detector and save them to hard disk as raw data. The raw data were then reconstructed into image slices using fan-beam filter-back projection (FBP) method. No data correction were performed during the tests reported in this paper.

In the following text, experiments carried out at the unit level were denoted as *test1* and experiments carried out at the system level were denoted as *test2* respectively. Detector unit's readout mode is set to mode 0 or float mode both in *test1* and *test2* setup.

### 2.3. Detector Performance Characterization Methods

#### 2.3.1. Noise Level

There are three sources of noises in the proposed detector unit: PDA noise, denoted as *n_p_*, which includes dark current from the photodiodes and thermal and electronic noises from the PDA circuitry, the circuit noise of the mDAS, denoted as *n_c_*, and the sampling noise of the ADAS1128, denoted as *n_a_*. Assume that *n_p_*, *n_c_* and *n_a_* are independent, the measured noise of the detector unit, denoted as *n* can be written as:

(1) $$\sigma_{n}^{2}=\sigma_{n_{d}}^{2}+\sigma_{n_{p}}^{2}+\sigma_{n_{a}}^{2}$$

where *σ* stands for the variance of the measurement. The sampling noise of the ADAS1128, *n_a_*, can be obtained from the manufacturer. When set to the float mode, the PDA will be disconnected from the detector, in which case *n_p_* is excluded and the measurement will only include *n_c_* and *n_a_*. So we can obtain two sets of measurements, one with *n_p_* and one without *n_p_*. Using these two measurement sets, *n_p_* and *n_c_* can be evaluated independently.

In readout mode 0 and float mode, 384 analog signals are sampled simultaneously from a single detector unit and the acquired *N* frames of data can be denoted as a 384-dimension vector series *X*(*n*)= [*x*_0_(*n*), *x*_1_(*n*),L, *x*_383_(*n*)]^T^. Noise level of the *i*'s channel is evaluated by its standard deviation, which defined as follows:

(2) $$\sigma_{i}={\left(\frac{1}{N}\sum_{n=0}^{N-1}{\;[x_{i}\;(n)-\mu_{i}]}^{2}\right)}^{1/2}$$

$$\mu_{i}=\frac{1}{N}\sum_{n=0}^{N-1}x_{i}\;(n)$$

When *N* is large enough, *σ_i_* is an excellent estimation of noise. Power spectrum of measurement is estimated through the Periodogram method. Actually, the Periodogram is the square of signal's DFT which defined as follows [4]:

(3) $$\begin{array}{cc} P_{i}\;(k)={\left|\;\overset{N-1}{\underset{n=0}{\text{∑}}}x_{i}\;(n)\cdot W^{nk}\;\right|}^{2}, & W=\text{exp}\;(-j\frac{2\pi }{N}) \end{array}$$

The experiments were carried out using a single detector unit with the setup described in Section 2.2. 96 frames of data were recorded with the detector unit set to float mode and mode 0 respectively. An integration time of 500 *us* was used and the X-ray was turned off in these tests. Table 2 lists the conditions for the noise tests.

#### 2.3.2. Independence

*Independence* refers to independence of different channels' response. K-L transform, also known as PCA (Principle Component Analysis), can be used to characterize the independence of different channels since it de-correlated components of *X*(*n*) as completely as possible [5,6]. This is achieved by analyzing the eigenvalues of *X*(*n*)'s covariance matrix *C*. More evenly distributed eigenvalues would suggest more independence between the channels. The covariance matrix is defined as:

(4) $$\begin{array}{cc} C=E\;[(X-\bar{X})\cdot {\left(X-\bar{X}\right)}^{T}], & \bar{X}=\frac{1}{N}\overset{N}{\underset{n=1}{\text{∑}}}X\;(n) \end{array}$$

where _E[·]_ denotes the expectation. Assume that matrix *C*'s eigenvalue vector is ʌ = [*λ*_1_, *λ*_2_, *λ*_384_]^*T*^ and *λ*_1_ ≥ *λ*_2_ ≥ *λ*_384_. PCA accumulative curve is defined as $f(k)=\overset{k}{\underset{i=1}{\text{∑}}}\lambda_{i}/\overset{N}{\underset{i=1}{\text{∑}}}\lambda_{i}.$ The ideal *f*(*k*) is a 45° straight line which goes through the origin point because all *λ_i_* are equal. In reality, however, *f*(*k*) is a convex curve because of the dependency between the channels.

Table 3 shows the five experiment cases that were carried out. Case 1 and case 2 would demonstrate independence of channels in float and mode 0. Case 3 to 5 are used to illustrate the utility of accumulative curve method since in cases 3 and 4, common X-ray flux noise and 50 Hz power noise are included respectively, which will affect all channels simultaneously and will impair independence quality considerably. These tests were also carried out using a single detector unit with the setup described in Section 2.2.

#### 2.3.3. Linearity and Sensitivity

In the experiments we checked two situations. In situation 1, tube voltage which was denoted as *U_tube_* and integration time which was denoted as *T* were fixed, whereas tube current which was denoted as *I_tube_* varied. In situation 2, *U_tube_* and *I_tube_* were fixed but *T* varied. The objective is to observe the linearity property with respect to *I_tube_* and *T*. Tables 4 and 5 show the experimental conditions for each situation.

Collected data were denoted as $x_{i}^{j}(n)$ , *n*=0,1,L,*N,i* is the index of channel *i*=1,2, 384 and *j* is the index of *T* or *I_tube_*. The linear model is showed as follows:

(5) $$\begin{array}{c} \begin{array}{cc} y=\beta_{i}x+{ɛ}_{i} & i=1,2,\text{L},384 \end{array}\\ {\bar{x}}_{i}=\frac{1}{N}\overset{N-1}{\underset{n=0}{\text{∑}}}x_{i}\;(n) \end{array}$$

where *y* is the response variable, *x* stands for *_T_* or *I_tube_*. Parameters of *β_i_* and *ε_i_* is got through linear regression by using least square method [7]. The mean value ${\bar{x}}_{j}=\frac{1}{N}\overset{N-1}{\underset{n=0}{\text{∑}}}x_{i}^{j}\;(n)$ is used as regressor value.

Sensitivity can be defined as:

(6) $$s_{i}\;(U_{tube})=\frac{\Delta R_{i}}{\Delta T\cdot \Delta I_{tube}}$$

where Δ*R*_i_ is channel's response increase, Δ*T* is integration time increase and Δ*I_tube_* is tube current increase. The physical meaning of sensitivity is the response increase of 1 mA increase of tube current and 1us increase of integration time. Actually in experiment, sensitivity can be get through two methods: ***Method 1:*** $s_{T}^{i}=\frac{\beta_{T}^{i}}{T}$ and ***Method 2:*** $s_{I}^{i}=\frac{\beta_{I}^{i}}{I_{tube}}$ and their results are expected to be equal. Actually, ***Medhod 1*** and ***Method 2*** are two ways to get channel *i*'s sensitivity, however, their results may be slightly different due to inevitable experiment error.

#### 2.3.4. Uniformity and Uniformity Correction

Due to the manufacturing process, the sensitivity of each channel in the detector unit may be different, that is *s_i_* ≠ *s_j_*, *i*≠*j*, which means for the same X-ray dose, response of different channel may be different. In order to evaluate the uniformity of sensitivity, we define the uniformity parameter *Uni* based on the responses.

Step1: acquire a frame of data, denoted as *x*(*i*),*i*=1,2, ,384;
Step2: *x*(*i*) is normalized to *x*ˆ(*i*)= *x*(*i*)/max *x*(*i*), where $\text{max}\;x(i\;)=\underset{1\leq i\leq 384}{\text{max}}\{x(i)\};$
Step3: calculate *Uni* by the following procedure:
  (7) $$\begin{array}{cc} Uni(U_{t})=\sqrt{\frac{1}{384}\overset{383}{\underset{i=0}{\text{∑}}}{\left(\hat{x}(i)-\bar{x}\right)}^{2}}, & \bar{x}=\frac{1}{384}\overset{383}{\underset{i=0}{\text{∑}}}\hat{x}\;(i) \end{array}$$

When the response is ideally uniform, *s_i_* are identical, so *Uni* = 0. For a chessboard image, however, we should get *Uni* = 0.5. Because of the non-uniform sensitivity, raw data collected from the detector unit should be corrected before reconstruction. Poor uniformity will introduce ring artifacts into reconstructed image. One possible solution is to tune different sensitivity *s* to an identical value. The correction procedure can be written as:

(8) $$R_{c}=\text{diag}\;(\frac{s_{\max}}{s_{1}},\frac{s_{\max}}{s_{2}},\cdots ,\frac{s_{\max}}{s_{384}})\cdot (Y-ɛ)$$

where *Y* is raw response vector, *ε* is the vector of *ε_i_* in linear model (7), *diag*(L) stands for a diagonal matrix and *R_c_* is the corrected data. Actually this procedure makes a simple linear projection, which tunes every channel's sensitivity to the maximum value among them, and therefore linear property of response is kept. The benefit of this transformation is that it makes full use of dynamic range. The drawback of the projection is that noise may be amplified as well. Considering the drawback of this procedure, a similar method may be used, which is:

(9) $$R_{c}=\text{diag}\;(\frac{s_{\min}}{s_{1}},\frac{s_{\min}}{s_{2}},\cdots ,\frac{s_{\min}}{s_{384}})\cdot (Y-ɛ)$$

where *s*_min_ is the minimum value of *s*_i_ The fact is that, however, the raw data is largely uniform and only a few channels are abnormal. Considering this fact, a more reasonable method is:

(10) $$\begin{array}{c} R_{c}=\text{diag}\;(\frac{\bar{s}}{s_{1}},\frac{\bar{s}}{s_{2}},\cdots ,\frac{\bar{s}}{s_{384}})\cdot (Y-ɛ)\\ \bar{s}=\frac{1}{384}\overset{384}{\underset{i=1}{\text{∑}}}s_{i} \end{array}$$

This method reconciled drawbacks of Equations (11) and (12) and may yield a probabilistic good result.

The former uniformity correction method is based on knowing each channel's sensitivity *s_i_* and *s_i_* varies with tube voltage *U_tube_* Physically, *U_tube_* determines the X-ray's energy spectrum and thus, the relationship between *s_i_* and *U_tube_* is non-linear. In order to gain an adaptive correction method, we should test the *s_i_*−*U_tube_* line through experiment and arbitrary *s_i_*(*U_tube_*) can be obtained through interpolation.

In the experiment, we used ***Method 1***, that is $s_{T}^{i}=\beta_{T}^{i}/T$ to test all channels' sensitivity $s_{T}^{i}$ Based on the results obtained, we corrected some randomly selected frames of data and a comparison of uniformity *Uni* between raw data and corrected data was shown. Finally, we showed a classical *s_i_*−*U_tube_* curve and the interpolated result. In order to show the *s_i_*−*U_tube_* line, we repeated the procedure of testing sensitivity $s_{T}^{i}$ in Section 2.3.3. We tested the response of 10, 32, 50 and 80 mA at each tube voltage. The tested tube voltage is 60, 70, 80, 90, 100, 110 and 120 kV. The interpolated data is at 115 kV.

## 3. Results and Discussion

### 3.1. Noise Level

**Figure 6.**
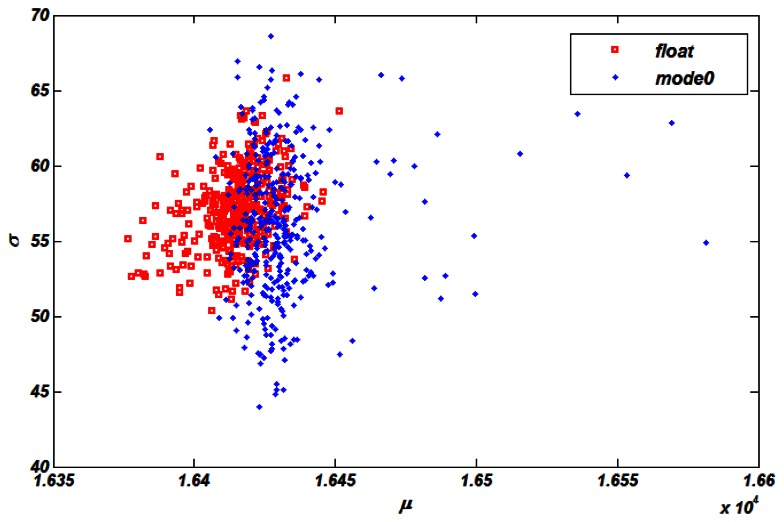
Noise level of float mode and mode0.

Figure 6 shows the noise level of each pixel by calculating *σ* and *μ* in Equation (2). During the calculation, *n_a_* was set to 60 for integration time 500 us, which was obtained from the manufacturer. Each point in the figure stands for a channel. Squares are channels tested in float mode, whereas the dots are channels tested in mode 0. These results show that the noise level in mode 0 and float mode meet the requirement since maximum standard variance max *σ*ˆ is under 70 and most of dots and squares concentrate near *σ* =55 In addition, mode 0's dots are dispersed wider in axis *σ* compared with that of float mode because of the introduction of PDA noise *n_p_* Furthermore, an obvious offset effect is introduced in axis *μ* Obviously, the dominating noise source is *n_a_*.

### 3.2. Channel Independence

**Figure 7.**
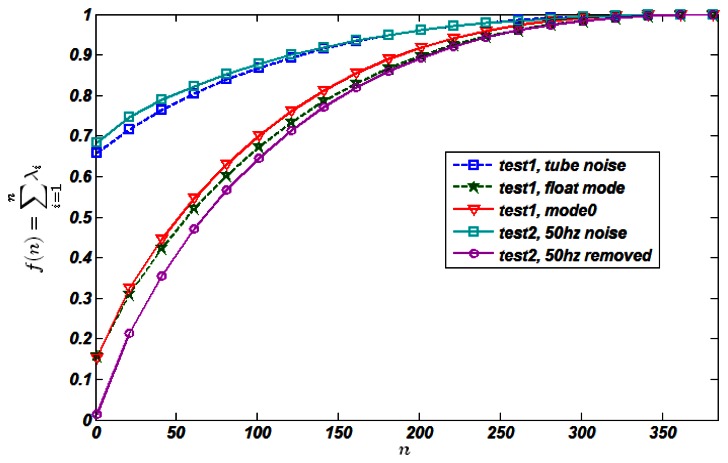
PCA accumulative curve.

Figure 7 shows the PCA accumulative curves for single unit tests (*test 1*) and CT detector test *(test 2)* respectively. Generally, upper accumulative curve's independence is poorer compared with the lower one. For example, the line of *test1*, *float mode* is lower than that of *test1*, *mode 0* since the introduction of *n_p_* to the latter, impairing the independence quality. Another phenomenon illustrates this property clearly is that when we removed the 50 Hz power noise from the raw data in *test2*, its accumulative line dropped dramatically, which is showed in Figure 7. The rationale of this phenomenon is that power noise acts as a common noise source which affects each channel simultaneously, in another word, channels are highly *dependent*. In order to further explain the utility of this method, we introduced another common noise source—X-ray tube noise *n_tube_* Usually *n_tube_* is significantly more conspicuous than that of *n_a_*+ *n_p_* + *n_c_* so that we can ignore the effects of the later when X-ray tube is turned on. In Figure 7, the *n_tube_* curve is also rather high, accord with our analysis and expectation.

Table 6 shows parameters related to the tests. *Samples* is the number of frame *N. Noise mean* is mean value of all channels noise level (*σ̄*. *First component ratio* is $\lambda_{1}/\overset{N}{\underset{i=1}{\text{∑}}}\lambda_{i}$ and it is important since it reveals the independence quality directly, the smaller the better. Test environment is some important test information.

### 3.3. Linearity

**Figure 8.**
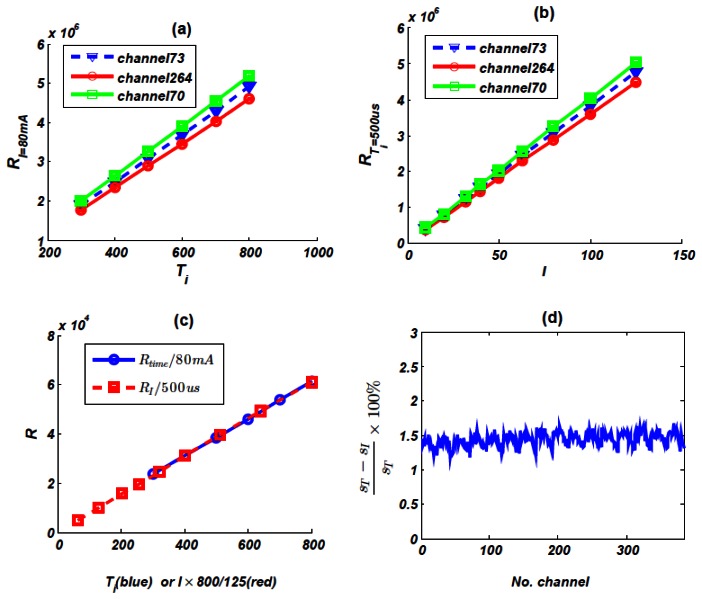
(**a**) Linearity property of *R*∼*T*, X-ray tube parameter is 90 kV, 80 mA; (**b**) Linearity property of *R* ∼ *I_tube_*, integration time *T* = 500 *us*; (**c**) Sensitivity property, red line's slope is *s_T_* and blue line's slope is *s_I_*; (**d**) Difference between *s_I_* and *s_T_*

In order to check the linearity of response of the detector unit, we randomly choose three channels and test its *R*∼*T* and *R* ∼ *I_tube_* line, *T* is integration time, *I_tube_* is tube current and *R* is the response. Figure 8 (a,b) shows *R*∼*T* and *R* ∼ *I_tube_* line respectively, which exhibit rather linear responses. Figure 8(c) shows channel 73's sensitivity that was got using the 2 different methods described in Section 2.3.3. The blue solid line's slope stands for *s_I_* and the red line stands for *s_T_*. Just as we have expected, they are almost the same. Figure 8(d) shows relative difference between *s_I_* and *s_T_* of each channel, the maximum difference is 1.67%.

### 3.4. Uniformity and Correction

**Figure 9.**
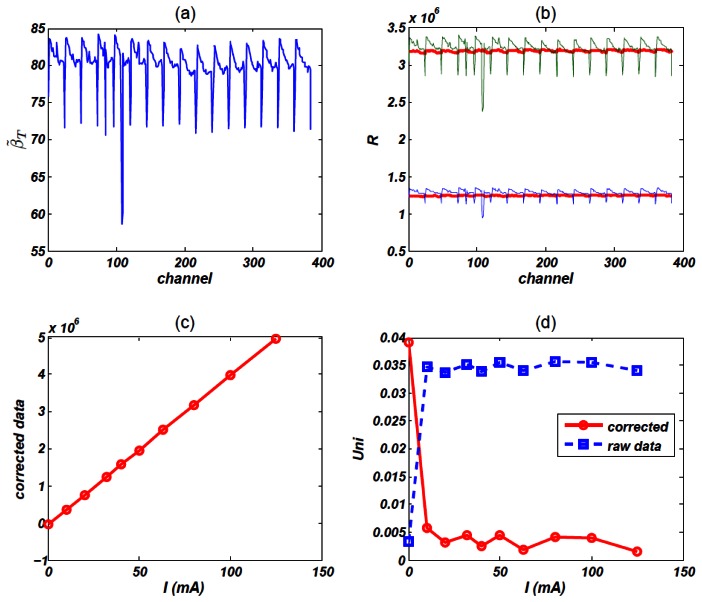
(**a**) Sensitivity *s_T_*. (**b**) Raw data and corrected results. The blue line is a frame data of 90 kV, 32 mA, green line is 90 kV, 80 mA, red lines are corrected results. (**c**) Corrected result of a randomly selected channel, obviously, this correction procedure keeps linearity. (**d**) Uniformity *Uni*, which is calculated by Equation (10). Red line is the corrected result, blue line is raw data's uniformity.

Firstly, we calculate the sensitivity *s_T_* of each channel (since *s_I_* is largely the same as *s_T_*, we plot *s_T_* here). Figure 9(a) shows the map of *s_T_*. From this picture, we can find dramatic *turbulence* of sensitivity, which reveals poor uniformity character of PDA assembly. It is obliged to correct raw data before reconstruction.

We try to correct some randomly selected frames of data by using the mean method of Equation (10), Figure 9(b) shows two results, which are two frames of data sampled at 90 kV, 32 mA and 90 kV, 80 mA respectively. Apparently, corrected data is far smoother than that of raw data.

Actually, this uniformity correction procedure is a simple linear transformation so that response's linearity is kept. Figure 9(c) demonstrated this analysis. We linked the corrected result of a channel directly with line segment and the whole line is just like a complete line segment. Table 7 shows channel 371's raw data and corrected data.

Finally, we show the a contrast of uniformity *Uni*, which was defined in Equation (7). For almost all response, the corrected result has better uniformity. For the zero input, however, the uniformity is undermined by the correction procedure, this is because raw data of zero input's uniformity is quite well, just as we have analyzed in noise level experiments.

Based on this fact, one solution is that if raw response is too small, near the zero-input response, for example, correction should not be performed. This strategy can avoid the obvious error introduced by correction procedure.

**Figure 10.**
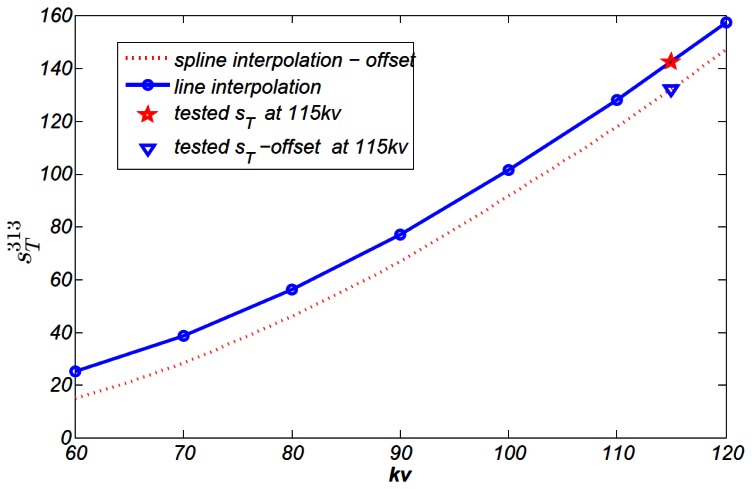
*s_T_* −*U_tube_* line of channel 313.

Figure 10 shows one classical *s_T_* −*U_tube_* line, which belongs to a randomly selected channel 313. Every channel has largely the same shape. We test sensitivity *s_T_* at X-ray tube voltage of 60 kV to 120 kV, covering clinical CT voltage range. Sensitivity at other voltage can be got through line or spline interpolation. The spline interpolated value at voltage 115 kV is 142.23 and tested value is 141.92, almost identical.

### 3.5. Reconstructed Images

**Figure 11.**
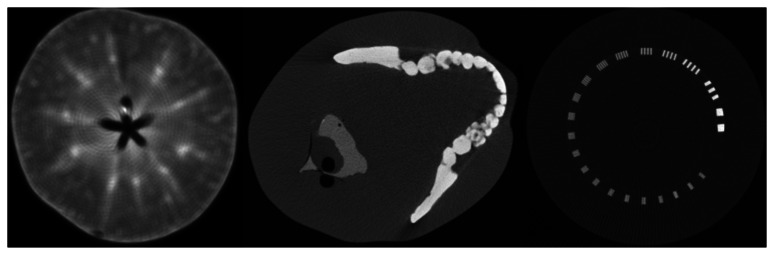
Reconstructed images of phantom. (left) slice of an apple; (middle) head phantom; (right) line pair phantom.

In whole CT detector test environment, we scanned some phantoms, such as an apple, a head phantom and a line pair phantom. The reconstruction method is fan beam FBP algorithm, without data correction. The result is showed in Figure 11. The general inner parts of these phantoms are clearly showed in the image except for some detailed information. Since lack of uniformity correction, circle-like artifacts is rather obvious, especially in apple image and line-pair image. Stripe artifact is conspicuous in head phantom image. Further analysis of image quality is beyond the scope of this article, we will demonstrate more analysis and result in the future articles.

## 4. Conclusions

In this article, we described a modular detector unit which can be easily tiled together to form a CT detector. Experiments were conducted to characterize the detector performance both at the single unit level and system level. The noise level, linearity and uniformity of the proposed detector unit were reported and results suggest that it is satisfactory for CT imaging. Initial imaging studies were also presented which demonstrated the potential application of the detector unit in actual CT scanners.

## Figures and Tables

**Table 1.** Properties of photodiode array assembly.

**Property**	**Condition**	**Value**
Photosensitivity (mA/W)	@510 nm	320 (typical)
Dark current	small pixel	10 pA
	large pixel	20 pA
Array size		24 × 24
Pixel pitch		0.95 mm/1.9 mm
Scintillator material		Gd_2_O_2_S

**Table 2.** Noise level experimental setup.

**Readout mode**	**Integration time *T* (*us*)**	**Frames *N***	**X-ray**
float	500	96	off
mode 0 (small pixel)	500	96	off

**Table 3.** Experiment cases of independence test.

**Case**	**Test environment**	**Frames N**	**Integration time** ***T (us)***	**Readout mode**	**X-ray**
1	*Test1*	476	500	float	Off
2	*Test1*	456	500	mode0	Off
3	*Test1*	468	500	mode0	90 kv,80 mA
4	*Test2*, 50 Hz power noise	512	500	mode0	Off
5	*Test2*, 50 Hz noise removed	472	500	mode0	Off

**Table 4.** Situation 1, *T* = 500 *us*, U_tube_ = 90 kV.

***I_tube_***	**10 mA**	**20 mA**	**32 mA**	**40 mA**	**50 mA**	**63 mA**	**80 mA**	**100 mA**	**125 mA**
frames N	72	72	72	72	72	72	72	72	72

**Table 5.** Situation 2, *I_tube_* = 80 mA, *U_tube_* = 90 kV.

***T***	**300 *us***	**400 *us***	**500 *us***	**600 *us***	**700 *us***	**800 *us***
frames N	72	72	72	72	72	72

**Table 6.** Independent test parameters.

	**Samples**	**Noise mean**	**First component ratio**	**Test environment**
***Test1*, float mode**	476	57.0	15.85%	0 kV, 0 mA, 500 *us*, float
***Test1*, mode0**	456	57.8	15.22%	0 kV, 0 mA, 500 *us*, mode0
***Test1*, quantum noise**	468	7,831.3	65.72%	90 kV,80 mA, 500 *us*, mode0
***Test2*, 50 Hz noise**	512	80.8	68.42%	0 kV, 0 mA, 500 *us*, mode0
***Test2*, 50 Hz removed**	472	60.7	1.37%	0 kV, 0 mA, 500 *us*, mode0

**Table 7.** Channel 371 s raw data and corrected data, 90 kV, 500 *us*.

*I_tube_* (mA)	0	10	20	32	40	50	63	80	100	125
Raw data 10^6^	0.016	0.414	0.808	1.302	1.626	2.034	2.577	3.262	4.066	5.053
Corrected 10^6^	−0.024	0.370	0.753	1.250	1.571	1.980	2.515	3.207	3.996	4.975
